# Induction of primordial germ cell-like cells from common marmoset embryonic stem cells by inhibition of WNT and retinoic acid signaling

**DOI:** 10.1038/s41598-023-29850-z

**Published:** 2023-02-23

**Authors:** Mayumi Shono, Keiko Kishimoto, Orie Hikabe, Masafumi Hayashi, Katsunori Semi, Yasuhiro Takashima, Erika Sasaki, Kiyoko Kato, Katsuhiko Hayashi

**Affiliations:** 1 Department of Stem Cell Biology and Medicine, Graduate School of Medical Sciences, Kyushu University, Higashi-ku, Fukuoka, 812-8582 Japan; 2 Department of Obstetrics and Gynecology, Graduate School of Medical Sciences, Kyushu University, Higashi-ku, Fukuoka, 812-8582 Japan; 3 Department of Marmoset Biology and Medicine, Central Institute for Experimental Animals, Kawasaki, 210-0821 Japan; 4 Department of Life Science Frontiers, CiRA, Kyoto University, Kyoto, 606-8507 Japan; 5 Department of Genome Biology, Graduate School of Medicine, Osaka University, Suita, Osaka 565-0871 Japan

**Keywords:** Germline development, Embryonic stem cells

## Abstract

Reconstitution of the germ cell lineage using pluripotent stem cells provides a unique platform to deepen our understanding of the mechanisms underlying germ cell development and to produce functional gametes for reproduction. This study aimed to establish a culture system that induces a robust number of primordial germ cell-like cells (PGCLCs) from common marmoset (*Callithrix jacchus*) embryonic stem cells. The robust induction was achieved by not only activation of the conserved PGC-inducing signals, WNT and BMP4, but also temporal inhibitions of WNT and retinoic acid signals, which prevent mesodermal and neural differentiation, respectively, during PGCLC differentiation. Many of the gene expression and differentiation properties of common marmoset PGCLCs were similar to those of human PGCLCs, making this culture system a reliable and useful primate model. Finally, we identified PDPN and KIT as surface marker proteins by which PGCLCs can be isolated from embryonic stem cells without genetic manipulation. This study will expand the opportunities for research on germ cell development and production of functional gametes to the common marmoset.

## Introduction

During early embryogenesis in mammals, primordial germ cells (PGCs), the origin of the germ cell lineage, are specified from the pluripotent cell population in response to BMP and WNT signals^1,2^, and then migrate into the gonads, which are the precursors of the ovary or testis formed at the dorsal wall of the coelom. During migration, PGCs undergo extensive DNA demethylation and reorganization of the histone modifications^2,3^. Recent studies focusing on early embryogenesis have illustrated species-specific differences in the embryonic tissue providing BMP and WNT signals for PGC specification. In mice, BMP4 and WNT3 are respectively supplied from the extraembryonic ectoderm (derived from trophoblast) and epiblast, whereas in cynomolgus monkeys, BMP4 and WNT3A are respectively supplied from the incipient amnion and cytotrophoblast^4,5^. In accordance with this difference in the source of BMP and WNT, the first appearance of PGCs in mouse embryos is at the posterior pluripotent epiblast, whereas that in cynomolgus monkeys is at the dorsal and later at the posterior side of the incipient amnion^6^. These results imply unknown species differences in subsequent germ cell development.

To elucidate such species differences, reconstitution of germ cell development using pluripotent stem cells in culture—namely, in vitro gametogenesis—is a powerful tool, especially for species for which early embryos are hardly available. Over the last decade, in vitro gametogenesis has been successfully employed to reconstitute PGC specification using embryonic stem cells (ESCs) and/or induced pluripotent stem cells (iPSCs) in mice, rat, cynomolgus monkeys, and humans^7–13^. In PGCs induced from ESCs and/or iPSCs, hereinafter PGC-like cells (PGCLCs), the gene expression profile and epigenetic modification recapitulate the properties of PGCs in vivo, allowing us to investigate genetic and epigenetic regulation in PGCs with a robust number of cells that are equivalent to early PGCs. Indeed, using the PGCLC system, transcription factors essential and sufficient for PGC specification have been isolated^9,14–19^, and the epigenetic landscapes in PGCs have also been elucidated in mice and humans^20–22^. Notably, the PGCLC system has uncovered species differences in the core gene-regulatory network evoking PGC specification, which is composed of *Blimp1*/*Prdm1*, *Prdm14* and *Tfap2c* in mice and *SOX17*, *BLIMP1*/*PRDM1* and *TFAP2C* in humans^23^. Since there are species differences in the PGC specification, the optimal culture conditions for robust induction of PGCLCs are not the same for all species. Indeed, there are even differences in the efficiency of PGCLC derivation among individual clones^12,24^. These aspects of the PGCLC system suggest that a custom-made culture system is required for induction of PGCLCs from ESCs/iPSCs in each species, and perhaps each cell clone.

The common marmoset (*Callithrix jacchus*), a New World monkey that can be bred in captivity, is a useful and reliable model for human development and aging, due to its relatively short gestation, small body, early sexual maturity, and short lifespan^25^. From the viewpoint of germ cell development, the short gestation and early sexual maturity of the common marmoset are extremely effective, as they allow the differentiation process and functionality of germ cells to be swiftly evaluated. Moreover, reproductive technologies have been sufficiently developed in the common marmoset to permit access to and manipulation of gametes and preimplantation embryos, and thereby the establishment of pluripotent stem cells and the creation of genetically modified animals^26–29^. Given the high time costs and ethical issues involved in using gametes and preimplantation embryos in humans and relatively large non-human primates, such as rhesus and cynomolgus macaques, the common marmoset would seem to be a convenient non-human primate for reproductive biology and medicine research. In this study, we aimed to establish a culture system that produces PGCLCs from common marmoset embryonic stem cells (cjESCs). Through multiple refinements, the culture system was optimized to produce a robust number of cjPGCLCs. The optimal condition showed that restriction of the somatic cell differentiation lineage though inhibition of RA and WNT signaling is a key process for the production of cjPGCLCs. This study should expand the opportunities for understanding PGC specification and in vitro gametogenesis in the common marmoset.

## Results

### Requirement of SOX17 and BMP4 signaling for differentiation of the germ cell lineage

**Figure 1:**
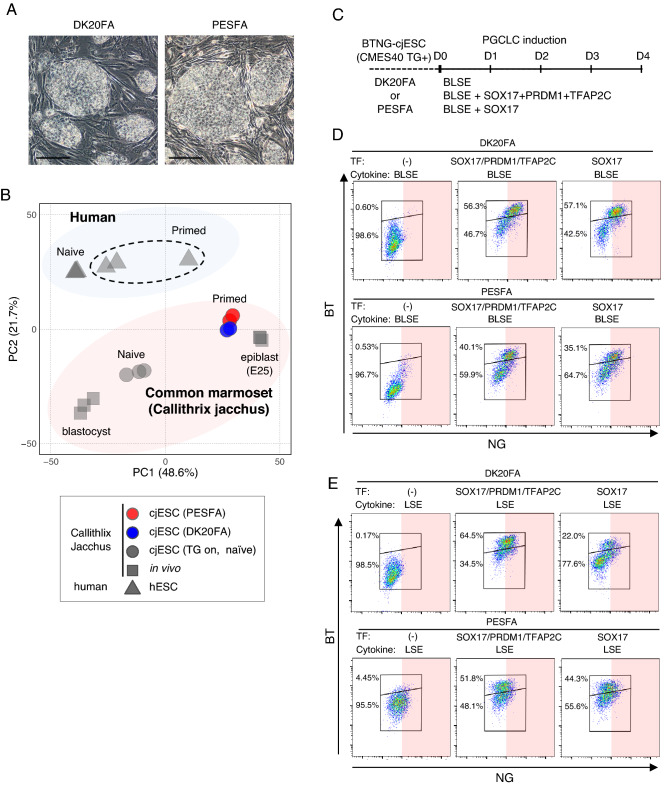
Induction of BTNG-positive cells by exogenous SOX17 expression with BMP4. (**A**) Morphology of cjESCs cultured on MEF in DK20FA and PESFA. Scale bar, 200 μm. (**B**) PCA of the gene expression profiles of pluripotent cell populations in common marmosets and humans. The genes used for the PCA were 839 ortholog genes annotated in both the common marmosets and humans, out of 886 differentially expressed genes between human naïve and primed ESCs^30^. The transcriptome profiles of common marmoset blastocysts, epiblasts and naïve ESCs were used^31^. (**C**) A schematic time course for PGCLC induction with or without exogenous SOX17 expression and BMP4. (**D**) FACS analysis of BTNG-positive cell induction with overexpression of transcription factors (TFs) and BLSE at day 4. (**E**) FACS analysis of BTNG-positive cell induction with overexpression of TFs and LSE at day 4.

We first investigated the optimal basal culture medium for maintenance of cjESCs (CMES40: 46XX)^26^ on mouse embryonic fibroblasts (MEFs) by comparing the results of culture using a conventional culture medium (KnockOut DMEM containing 20% KSR, bFGF and activin A; hereinafter DK20FA) and using an alternative culture medium (Primate ESC medium containing bFGF and activin A; hereinafter PESFA). Under both conditions, cjESCs formed colonies with a flat appearance (Fig. 1A) and expressed pluripotent transcription factors such as POU5F1, NANOG and SOX2, and surface marker proteins associated with pluripotency such as TRA1-60, SSEA4 and integrin α6 (Supplementary Fig. 1A). cjESCs propagated biexponentially for at least 25 days under both conditions, but the proliferation rate was greater in PESFA than in DK20FA (Supplementary Fig. 1B). We determined the transcriptomic profiles of cjESCs by RNA sequence analysis (RNA-seq) and then compared them with the transcriptomic profiles of naïve and primed human ESCs^30^, transgene-induced naïve cjESCs^31^, common marmoset blastocysts, and epiblast at embryonic day (E) 25 by principal component analysis (PCA). This analysis revealed that cjESCs under both conditions correspond to the primed state (Fig. 1B).

To facilitate the evaluation of PGCLC differentiation, we established a reporter cjESC line by inserting the *tdTomato* and *eGFP* genes into the *BLIMP1/PRDM1* and *NANOS3* gene loci, respectively, both of which are conserved marker genes for PGC specification^9,10,32,33^ (Supplementary Fig. 1C). Using these reporter cjESCs, hereinafter *BLIMP1*-*tdTomato* (BT) and *NANOS3-EGFP* (NG) cjESCs, we evaluated the competence of PGCLC differentiation by overexpressing a set of transcription factors, *SOX17, PRDM1 and TFAP2C*, and/or culturing with a set of cytokines consisting of BMP4, LIF, SCF and EGF (BLSE), any one of which is sufficient for PGCLC induction in human ESCs/iPSCs^9–11^ (Fig. 1C). Unlike in the induction of human PGCLCs, however, culturing with the set of cytokines alone yielded no BTNG-positive cells. But when the transcription factors were expressed in the reporter cjESCs cultured with the cytokines, a robust induction of BTNG-positive cells was observed (Fig. 1D). Moreover, with the set of cytokines, overexpression of *SOX17 *alone was sufficient for induction of BTNG-positive cells (Fig. 1D). There was no clear difference between ESCs maintained in DK20FA and those maintained in PESFA. In the absence of BMP4 (hereinafter the LSE condition), overexpression of *SOX17*, *PRDM1* and *TFAP2C* or *SOX17* alone still induced BTNG-positive cells, but the expression level of NG was lower than that in the BTNG-positive cells induced under the BLSE condition (Fig. 1E). These results demonstrated that BMP4 signaling and overexpression of SOX17 are both required for the induction of BTNG-positive cells from cjESCs.

### Dissection of molecular pathways downstream of SOX17 and BMP4

**Figure 2:**
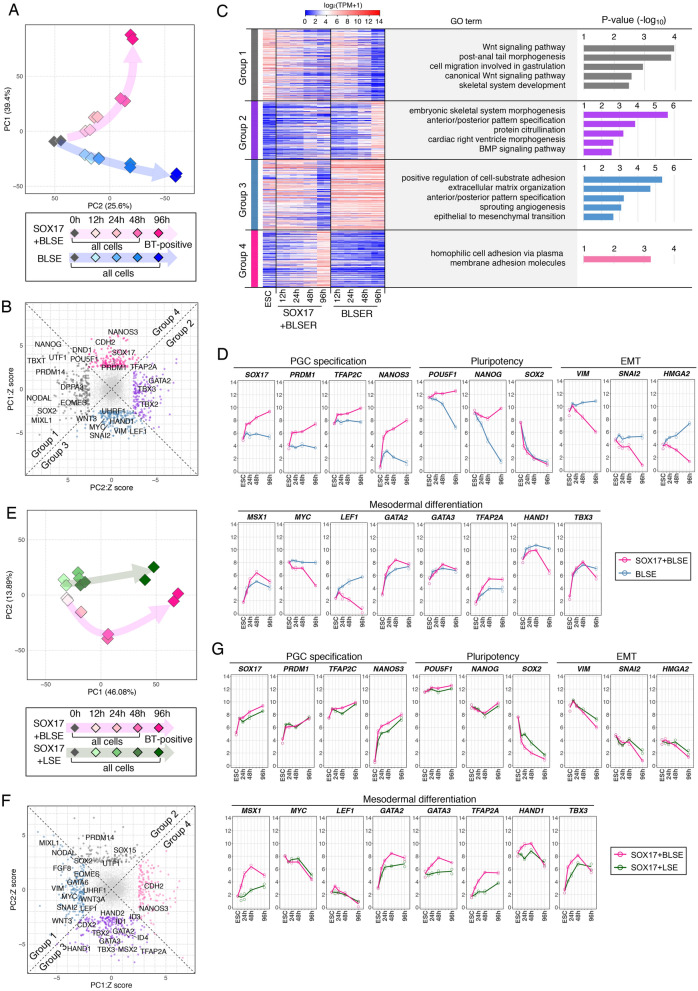
Dissection of molecular pathways downstream of SOX17 and BMP4. (**A**) Differentiation trajectories during the induction of BTNG-positive cells with SOX17 + BLSE and BLSE alone. Shown are PCA plots of cjESCs and cells at 12, 24, 48, or 96 h of culture under the condition indicated. In this series of analyses, cjESCs were maintained under the DK20FA condition. (**B**) A scatterplot of the Z scores of the genes for the PC1 and 2 axes. The scatterplot was made using the Z scores of the genes for the PC1 and 2 axes shown in (**A**). Genes with > 2.5 SDs (695 genes) are shown. (**C**) Heatmap and GO analyses of genes contributing to each PC. The heatmap shows the expression level of genes categorized in the Z score plot in (**B**). Representative GO terms for each gene group and their p-values are shown at the right. (**D**) Expression dynamics of representative genes during the induction of BTNG-positive cells with SOX17 + BLSE and BLSE alone. The graphs show the expression levels of genes involved in PGC specification, pluripotency, epithelial-mesenchymal transition (EMT) and mesoderm differentiation during the induction of BTNG-positive cells. Each plot is based on duplicated RNA-seq analyses. The mean values are connected by the line. (**E**) Differentiation trajectories during the induction of BTNG-positive cells with SOX17 + BLSE and SOX17 + LSE. (**F**) A scatterplot of the Z scores of the genes for the PC1 and 2 axes. The scatterplot was made using the Z scores of the genes for the PC1 and 2 axes shown in (**E**). Genes with > 2.5 SDs (651 genes) are shown. (**G**) Expression dynamics of representative genes during the induction of BTNG-positive cells with SOX17 + BLSE and SOX17 + LSE. Details are the same as in (**D**). The analyses in this figure were performed using the common genes between marmosets and humans (15,755 genes).

To elucidate the molecular pathways of SOX17 and BMP4 involved in the PGCLC induction, we examined the transcriptomic trajectory from cjESCs to BTNG-positive cells under BLSE with or without SOX17 by RNA-seq analysis (Fig. 2A and Supplementary Fig. 2A). PCA illustrated two distinct trajectories: a BLSE-driven trajectory along the PC2 axis and a SOX17 + BLSE-driven trajectory along the PC1 axis (Fig. 2A). Indeed, genes positively contributing to PC1 of the PCA, including *PRDM1* and *NANOS3,* were associated with PGC specification (Fig. 2B, Group 4). In contrast to the genes in Group 4, those in Group 3, including *SNAI2*, *VIM*, *HAND1* and *LEF1*, were associated with epithelial–mesenchymal transition (EMT) and mesodermal differentiation (Fig. 2B). Gene ontology (GO) analysis of the genes in Group 3 showed enrichment of the GO terms related to EMT and mesodermal differentiation (e.g., anterior/posterior pattern specification and sprouting angiogenesis) (Fig. 2C), indicating that cjESCs readily differentiate to mesodermal cells in the absence of SOX17. In line with this expectation, genes negatively contributing to the PC2 axis included genes downstream of WNT and BMP signals, such as *TBX2*, *TBX3* and *GATA2* (Fig. 2B, Group 2), and GO analysis showed enrichment of the GO terms related to mesodermal differentiation (e.g., embryonic skeletal system morphogenesis and anterior/posterior pattern specification) (Fig. 2C, Group 2). Genes in the Group 1, the opposite group to the Group 2, were pluripotency-associated genes, such as *NANOG*, *SOX2* and *PRDM14* (Fig. 2B). Indeed, analysis of the expression of representative genes in cells under the SOX17 + BLSE condition showed not only upregulation of PGC marker genes but also constant expression of pluripotency-associated genes, such as *POU5F1* and *Nanog*, and downregulation of the genes involved in EMT (Fig. 2D, pink lines). In contrast, cells cultured under the BLSE condition without *SOX17* did not show upregulation and maintenance of PGC- and pluripotency-associated gene expression, respectively, and instead showed upregulation of some somatic genes, such as *LEF1* and *HAND1* (Fig. 2D, blue lines). These results suggested that a critical role of SOX17 is to trigger the expression of PGC-associated genes and maintain the expression of pluripotency-associated genes with suppression of a somatic cell differentiation program initiated by EMT.

To clarify the effect of BMP4, we next compared the transcriptomic trajectory between the SOX17 + BLSE and SOX17 + LSE conditions. The difference in the transcriptomic trajectory, though subtle, was represented by the PC2 axis (Fig. 2E). Genes negatively contributing to the PC2 axis included *MSX2, ID1*, *ID4*, *GATA2* and *GATA3*, all of which are direct targets of BMP signaling^19,34^ (Fig. 2F, Group 3). Two of these genes, *GATA2* and *GATA3*, are cooperatively involved in human PGCLC differentiation^19^. It is noteworthy that *TFAP2A* is found in the genes negatively contributing to the PC2 axis (Fig. 2F, Group 3). *TFAP2A* is preferentially expressed in the nascent amnion containing PGC precursors in cynomolgus monkeys^6^ and is also expressed in the precursor population during PGCLC induction from human ESCs^18,35^. The expression of representative genes confirmed the upregulation of *GATA2, GATA3* and *TFAP2A* (Fig. 2G, pink lines). These results suggested that BMP-mediated signaling promotes commitment to the precursor cell population through induction of *GATA2*, *GATA3* and *TFAP2A*. Of note, the expressions of PGC specification-related genes, pluripotency-associated genes, and EMT-related genes were not affected in absence of BMP4 (Fig. 2G), suggesting that WNT signaling has a prominent role in somatic cell differentiation during PGC specification.

### Repression of WNT signaling enabled the induction of BTNG-positive cells

**Figure 3:**
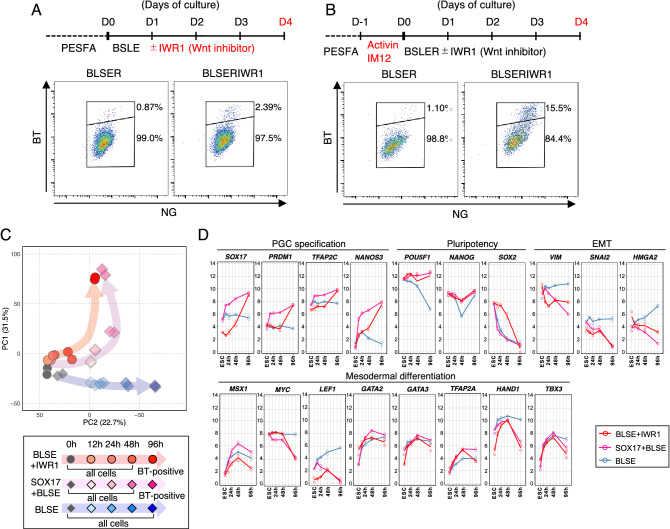
Efficient induction of BTNG-positive cells in the presence of IWR1. (**A**) Enhanced induction of BTNG-positive cells with IWR1. Shown are the schematic time course of the BTNG-positive cell induction with IWR1 (top) and a representative FACS plot of cells at day 4 of induction (bottom). (**B**) Further enhancement of the BTNG-positive cell induction with the preculture period and IWR1. Shown are a schematic time course of the BTNG-positive cell induction with the preculture period and IWR1 (top) and a representative FACS plot of cells at day 4 of induction (bottom). (**C**) Differentiation trajectories during the induction of BTNG-positive cells with BLSE + IWR1. Shown are PCA plots of cjESCs and cells at 12, 24, 48, or 96 h of culture under the condition indicated. The differentiation trajectories under the conditions with SOX17 + BLSE and BLSE alone were the same as in Fig. 2A. (**D**) Expression dynamics of representative genes during the BTNG-positive cell inductions with BLSE + IWR1, SOX17 + BLSE and BLSE alone. Graphs show the expression levels of genes involved in PGC specification, pluripotency, EMT and mesodermal differentiation. Each plot is based on duplicated RNA-seq analyses. The mean values are connected by a line. The analyses in this figure were performed using the common genes between marmosets and humans (15,755 genes).

Given that an important effect of SOX17 is to prevent somatic cell differentiation triggered by activation of WNT signaling during PGCLC differentiation, an appropriate inhibition of the signaling by small chemicals would enable differentiation of BTNG-positive cells without exogenous SOX17 expression. To test this possibility, we added IWR1, an inhibitor of Wnt signaling, to BLSE (Fig. 3A). Under this condition, a small but distinct population of BTNG-positive cells was observed at 96 h of culture in the presence of IWR1 (Fig. 3A). This result indicates that WNT is no longer required for cjPGCLC induction in BLSE. As it is known that WNT signaling is required to confer competence for PGC differentiation^14,17^, we temporally added a WNT agonist, IM12, and activin A to the medium one day before cjPGCLC induction (Fig. 3B). This pre-treatment clearly enhanced the induction of BTNG-positive cells. These results indicate that temporal controls of WNT signaling, activation in the pre-treatment and inhibition in PGCLC differentiation, are important for the induction of BTNG-positive cells. This condition with IM12 addition as part of the pre-treatment and IWR1 addition for the induction of BTNG-positive cells is described hereinafter as the BLSE + IWR1 condition.

The gene expression profile of BTNG-positive cells at 96 h of culture under the BLSE + IWR1 condition with one-day of pre-treatment was similar to that of BTNG-positive cells induced by SOX17 + BLSE (Fig. 3C, Supplementary Fig. 2B and 3B). Looking at the transcriptomic trajectory, addition of IWR1 prevented a trend toward differentiation, represented by the PC2 axis, at 48 h of culture (Fig. 3C). It was noteworthy that the expression of pluripotency-associated genes, including *POU5f1* and *NANOG*, was maintained with IWR1, and the expression of EMT-related genes was downregulated (Fig. 3D, red lines). In contrast to the rapid upregulation of PGC marker gene expression under the SOX17 + BLSE condition, the expression of such genes was gradually upregulated in the cells cultured with BLSE + IWR1 condition (Fig. 3D). This observation is consistent with previous findings that an excessive activation of WNT signaling perturbs PGCLC differentiation in mice and humans^14,17^. Thus, we conclude that inhibition of WNT signaling promotes the induction of BTNG-positive cells by repressing the somatic differentiation and maintaining pluripotency-associated gene expression, by which PGC competence would be properly conferred on the cells.

### Repression of retinoic acid signaling enhanced the induction of BTNG-positive cells

**Figure 4:**
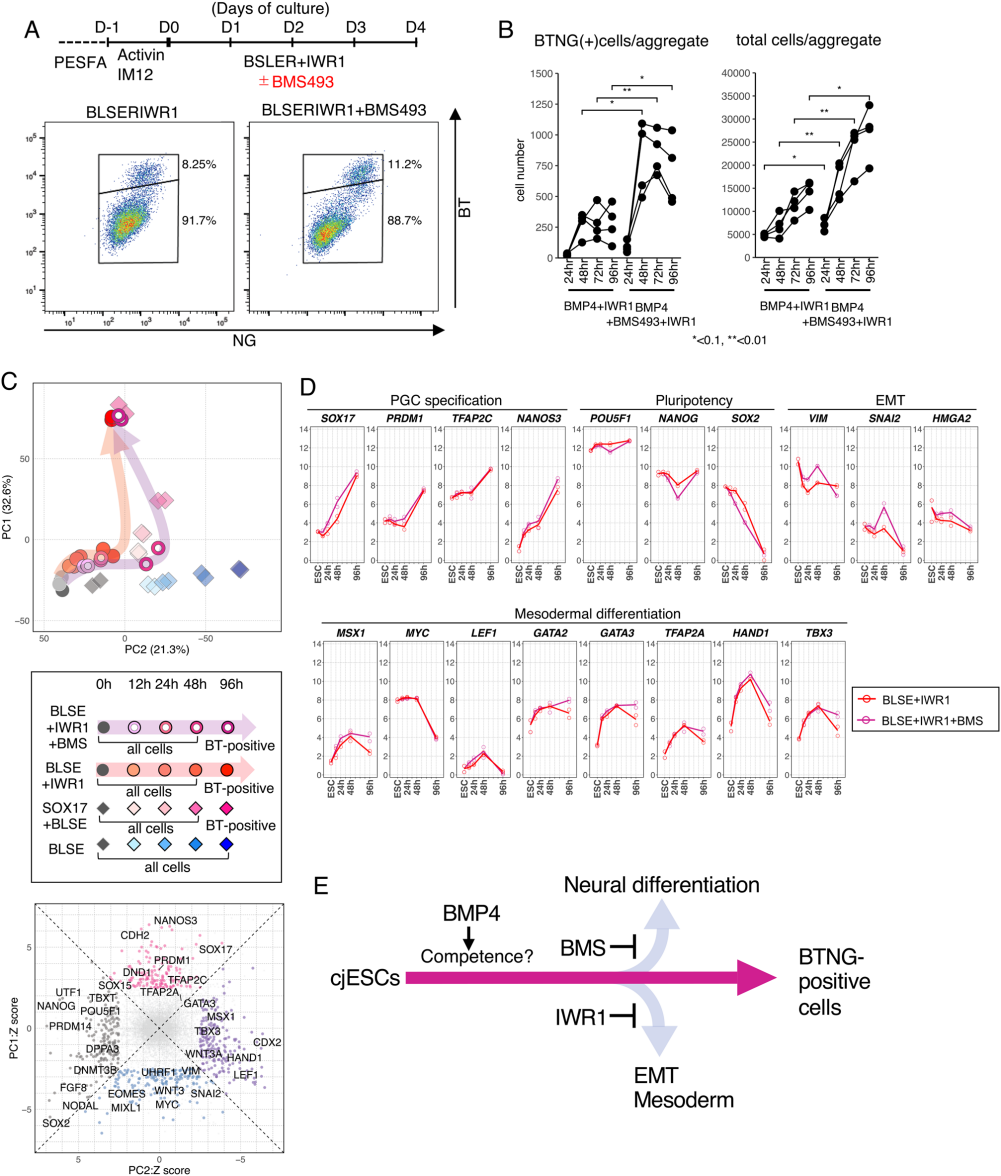
A robust induction of BTNG-positive cells with IWR1 and BMS493. (**A**) A robust induction of BTNG-positive cells with BLSE + IWR1 + BMS. The top panel shows the schematic time course of the BTNG-positive cell induction with IWR1 and BMS493 after preculture, and the bottom panel is a representative FACS plot of cells at day 4 of induction. (**B**) The numbers of cells in the induction of BTNG-positive cells. Shown are the numbers of BTNG-positive cells (left) and total cells per aggregate (right). The plots were based on experiments repeated 4 times. Each line traces values obtained in a separate series of experiments. (**C**) Comparison of the differentiation trajectories under the BLSE + IWR1 + BMS condition. The top plot shows the PCA of cjESCs and derivatives cultured under the condition shown below. The bottom scatterplot shows the Z scores of the genes for the PC1 and 2 axes. Genes with > 2.5 SDs (690 genes) are shown. (**D**) Expression dynamics of representative genes during the induction of BTNG-positive cells under the BLSE + IWR1 + BMS condition in comparison with those of representative genes during the induction of BTNG-positive cells with BLSE + IWR1. Graphs show the expression levels of genes involved in PGC specification, pluripotency, EMT and mesodermal differentiation. Each plot is based on duplicated RNA-seq analyses. The mean values are connected by a line. The analysis was performed using the common genes between marmosets and humans (15,755 genes). (**E**) A schematic diagram of signaling effects on the differentiation from cjESCs to BTNG-positive cells.

Despite the finding that WNT inhibition exerted a positive effect on induction of BTNG-positive cells, we found a slight enrichment of gene expression related to neuronal development, as this enrichment was represented by the GO terms (e.g., central nervous system myelination and synaptic transmission, glutamatergic) in the transcriptome at 48 h of culture in BLSE + IWR1 (Supplementary Fig. 3A). In addition, the expression of genes related to mesodermal tissue development was reduced. This could be a side effect of WNT inhibition that induces anterior specification during gastrulation^36^. As a possible means of preventing the activation of this neuronal program, we evaluated the effect of inhibiting the retinoic acid signal that promotes differentiation into the ectoderm lineage in mouse and human pluripotent stem cells^37,38^. By adding BMS493, a pan-retinoic acid receptor inverse agonist, to BLSE + IWR1 (hereinafter the BLSE + IWR1 + BMS condition), the efficiency of BTNG-positive cell induction was improved, especially with respect to the number of cells yielded (Fig. 4A,B). In addition, the total number of cells in the aggregates was also increased (Fig. 4B). The transcriptome of BTNG-positive cells at 96 h of induction under the BLSE + IWR1 + BMS condition was similar to that of BTNG-positive cells induced without BMS493 (Fig. 4C, Supplementary Figs. 2C and 3B). In contrast, the effect of BMS493 was obvious at 48 h of culture: the transcriptomic profile shifted in a negative direction on the PC2 axis, and this shift represented the WNT-mediated differentiation (Fig. 4C). The expression dynamics of representative genes during the induction showed a slight enhancement of WNT signaling, as represented by the upregulation of EMT-related gene expression and downregulation of pluripotency-associated gene expression (Fig. 4D, plum lines). These results indicate that addition of BMS493 enhanced WNT-signaling to some extent, and the WNT-signaling in turn induced posteriorization during gastrulation. Taken together, these results showed that the fine tuning of WNT signaling and retinoic acid signaling resulted in BTNG-positive cell differentiation from cjESCs through the inhibition of EMT-mediated mesodermal differentiation and the inhibition of neural differentiation, respectively (Fig. 4E).

### Evaluation of epigenetic modifications and differentiation potential of BTNG-positive cells

**Figure 5:**
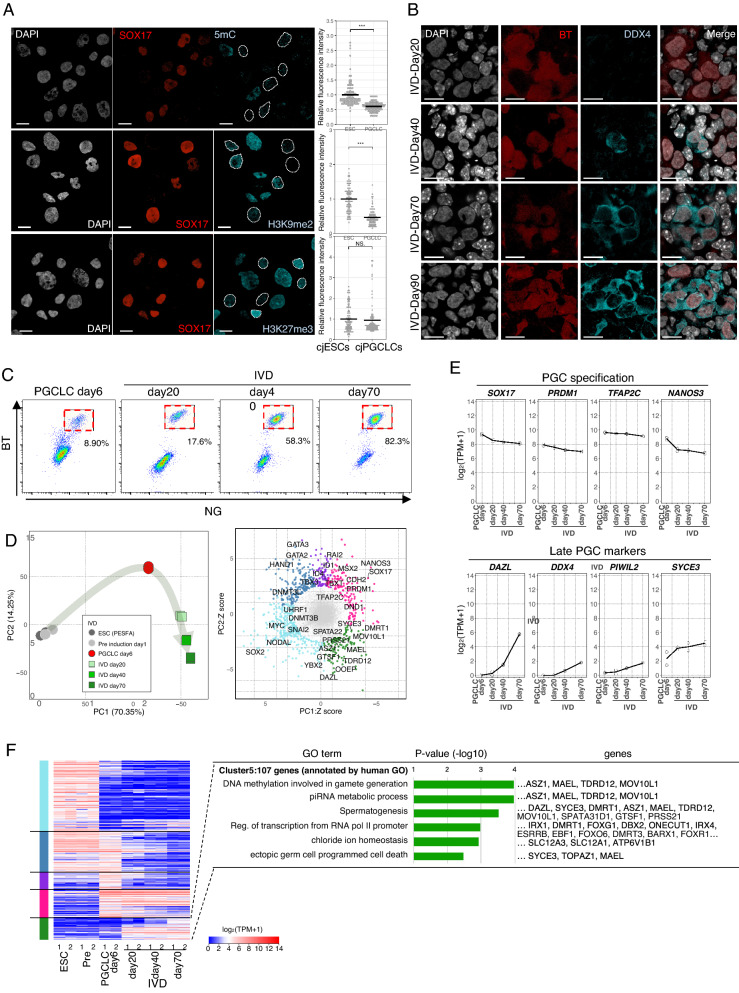
Epigenetic and differentiation properties of BTNG-positive cells. (**A**) Immunofluorescence analyses of representative epigenetic markers in BTNG-positive cells. Images show the results of immunofluorescence analysis of H3K9me2, H3K27me3 and 5-methyl cytosine (5mC) with DAPI in SOX17-positive cells at day 4 of culture under the BLSE + IWR1 condition. Plots on the right show relative fluorescence intensities, in comparison to those in cjESCs, as determined using Image J software. The average fluorescence intensity of cjESCs was set as 1. ***P < 0.001 (using Welch’s t test). NS, not significant. Scale bar, 20 μm. (**B**) Expression of DDX4 in the reaggregations with mouse gonadal somatic cells. Images show the results of immunofluorescence analysis of DDX4 with DAPI in BT-positive cells at days 20, 40, 70, and 90 of culture in the xenogeneic reconstituted ovaries. Scale bar, 20 μm. (**C**) FACS analysis of BTNG-positive cells in the xenogeneic reconstituted ovaries. (**D**) Differentiation trajectories of BTNG-positive cells in the xenogeneic reconstituted ovaries. Shown are PCA plots of cjESCs before or after the preculture, BTNG-positive cells at day 6 of induction, and BTNG-positive cells in the xenogeneic reconstituted ovaries at the day indicated. The scatterplot at right shows the Z-normalized loading scores of the genes contributing to the PC1 and PC2 axes. Genes with a radius > 2.5SDs are shown (887 genes), and are colored according to the gene classification in (**F**). The key genes are annotated in the plot. (**E**) Expression dynamics of representative genes in BTNG-positive cells cultured in the xenogeneic reconstituted ovaries. Each plot is based on duplicated RNA-seq analyses. The mean values are connected by a line. (**F**) Heatmap and GO analyses of genes upregulated in the xenogeneic reconstituted ovaries. The heatmap shows the expression levels of 887 genes highly contributing to the PC1 and PC2 axes, which are shown in (**D**). The genes were sorted by unsupervised hierarchical clustering, which provided 5 clusters according to the expression dynamics. Representative GO enrichments with *p* values and key genes in the cluster (green) characterized by gene expression upregulated in the xenogeneic reconstituted ovaries are shown. The colors at the left correspond to those in the Z score plot in (**D**). The analysis was performed using the common genes between marmosets and humans (15,755 genes).

To further characterize BTNG-positive cells, we next examined their epigenetic properties. In mice, cynomolgus monkeys and humans, PGC(LC)s have unique epigenetic properties—namely, a low level of CpG methylation and H3K9 di-methylation (H3K9me2), and a high level of H3K27 tri-methylation (H3K27me3)^7,9,10,21,22,39^. Consistent with these conserved properties, immunofluorescence analysis revealed that BTNG-positive cells at day 4 of induction under a BLSE + IWR1 condition possessed significantly lower levels of CpG methylation and H3K9me2 in the genome (Fig. 5A). In contrast, the level of H3K27me3 in BTNG-positive cells was not significantly different from that in cjESCs. These trends in the epigenetic properties were reproduced in BTNG-positive cells induced under the BLSE + IWR1 + BMS condition (Supplementary Fig. 4A). The insufficient decrease in the H3K27 may have been due to the very high level of H3K27me3 in cjESCs and/or to the developmental stage of BTNG-positive cells being too early to raise the level of H3K27me3. Collectively, these results indicate that genome-wide epigenetic reprogramming occurs in BTNG-positive cells.

To evaluate the potential of BTNG positive cells for use in studying the germ cell lineage, reaggregation with gonadal somatic cells would be effective^40,41^. Because there are only limited sources of common marmoset gonads at an appropriate developmental stage, we used mouse female embryonic gonadal somatic cells at E12.5 for reaggregation with BTNG-positive cells at day 6 of induction under the BLSE + IWR1 condition (Supplementary Fig. 4B). Upon reaggregation, BTNG-positive cells started to proliferate and to express *DDX4*, a conserved marker of later PGCs^42^ (Fig. 5B). During the culture, nuclear staining with DAPI become faint in BTNG-positive cells, suggesting that the epigenetic reprogramming had progressed. A similar differentiation was observed in the reaggregates made using BTNG-positive cells induced under the BLSE + IWR1 + BMS condition (Supplementary Fig. 4C). BTNG-positive cells were viable in the aggregates until at least 90 days of culture (Fig. 5B,C, Supplementary Fig. 4C ). During this period, the transcriptomic profiles began to include the expression of later PGC marker genes, such as *DAZL*, *PIWIL2*, *MAEL* and *SYCE3* (Fig. 5D,E). GO analysis of the genes enriched in BTNG-positive cells in the reaggregates exhibited the GO terms (e.g., DNA methylation involved in gamete generation, piRNA metabolic process and Spermatogenesis), reinforcing that germ cell development proceeded in BTNG-positive cells upon reaggregation with mouse gonadal somatic cells (Fig. 5F). These findings, together with the transcriptomic properties, led us to conclude that the BTNG-positive cells could be defined as PGCLCs derived from cjESCs, and we therefore designated them cjPGCLCs.

### Surface markers for cjPGCLCs and clonal variation in differentiation potential

**Figure 6:**
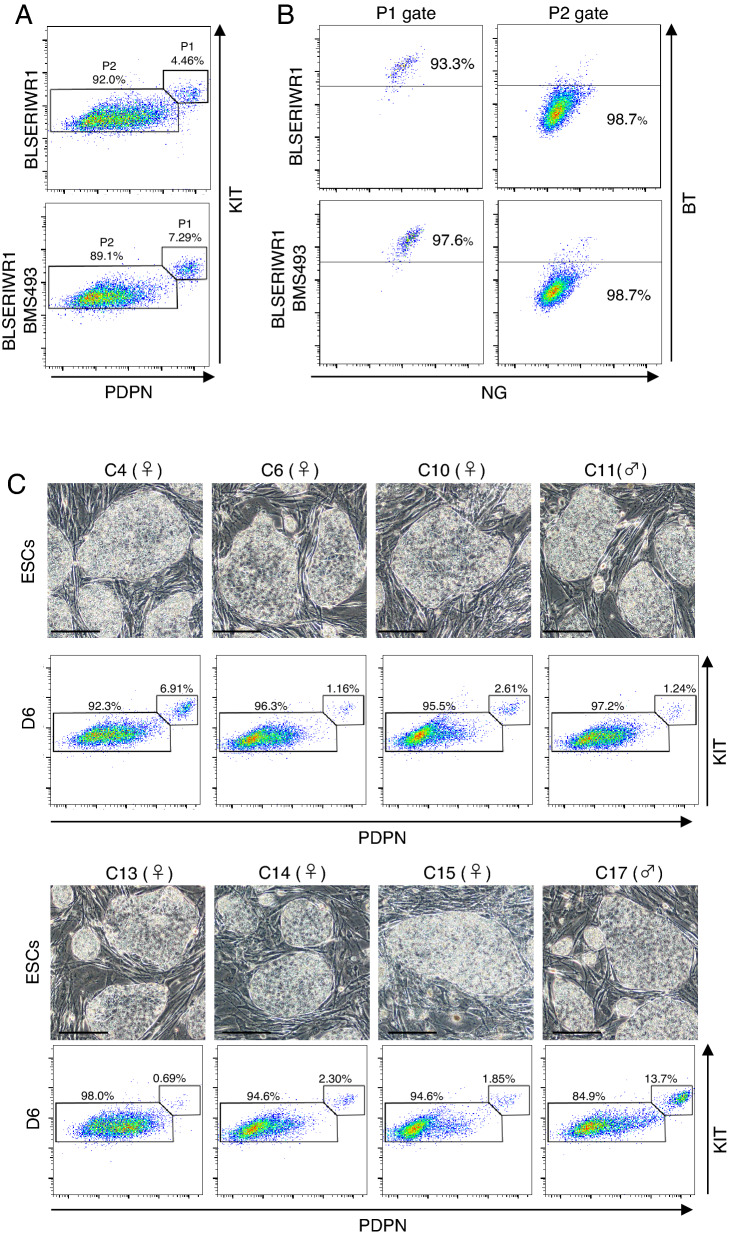
Induction and isolation of cjPGCLCs from non-reporter cjESC lines. (**A**) Isolation of non-reporter cjPGCLCs with surface marker proteins. FACS plots show the expression of PDPN and KIT in cells at day 6 of culture under a BLSE + IWR1 or BLSE + IWR1 + BMS condition. Note that a separable cell population was observed in the double-positive fraction. (**B**) Validation of cells double-positive for PDPN and KIT. FACS plots show BTNG expression in the cell populations gated in P1 and P2, as shown in (**A**). Note that more than 90% of cells double-positive for PDPN and KIT are positive for BTNG. (**C**) Induction of cjPGCLCs from multiple cjESC lines. The images show the morphology of each cjESC line cultured on MEF in PESFA. FACS plots below the images show the expression of PDPN and KIT in cells at day 6 of culture under the BLSE + IWR1 + BMS condition. Scale bar, 200 μm.

Because the requirement of reporter constructs limits the applicability of cjPGCLCs, we next explored surface marker proteins that discriminate cjPGCLCs from other cell types. Based on the relevant reports, transcriptomic analysis and availability of antibodies, we chose PODOPLANIN (PDPN), KIT and INTEGRINa6 (ITGA6) as candidates. FACS analysis using specific antibodies revealed that the expressions of PDPN and KIT were preferentially detected in BT-positive cells induced by exogenous *SOX17* expression in BLSE (Supplementary Fig. 5A). Consistent with this finding, transcripts of *PDPN* and *KIT* were enriched in cells expressing a higher level of BLIMP1 (Supplementary Fig. 5B). Simultaneous staining with KIT and PDPN antibodies illuminated a distinct cell population that is nearly identical to BTNG-positive cells at day 6 of induction: more than 90% of cells double-positive for KIT and PDPN were positive for BTNG (Fig. 6A,B). These results demonstrated that KIT and PDPN are markers available for the identification of cjPGCLCs without reporter gene constructs.

Next, we used these surface marker proteins to evaluate the clonal variation of cjESCs in the efficiency of PGCLCs induction. To test this variation, we used eight previously established lines of cjESCs^29^. These cjESCs proliferated biexponentially under the PESFA condition while maintaining sharp-edged and tightly packed colonies (Fig. 6C and Supplementary Fig. 5C). Induction of cjPGCLCs under the BLSE + IWR1 + BMS condition revealed clonal variation in the efficiency ranging from 0.7% up to 14% among these cjESC lines (Fig. 6C). Although the number of cjESC clones was not sufficient, one of the two male cjESC lines showed PGCLC differentiation greater than 10%, but none of the six female cjESC lines showed such efficiency. This clonal variation and possible sex-bias in the competence of PGC differentiation are reminiscent of the clonal variation of human and cynomolgus monkey pluripotent stem cells in the efficiency of PGCLC induction^12,24^. These results demonstrated that simultaneous staining for KIT and PDPN is available for the detection of cjPGCLCs induced from various cjESCs without the reporter constructs.

## Discussion

In this study, we developed a culture system to produce cjPGCLCs from cjESCs. The culture system was largely consistent with PGCLC differentiation in humans and cynomolgus monkeys^9–12^. For example, SOX17, a key transcription factor for PGCLC induction in primates but not in mice, plays a critical role in the cjPGCLC system. A robust number of cjPGCLCs were induced by exogenous *SOX17* expression in the presence of BMP4, whereas *SOX17* expression alone was not sufficient for the PGCLC induction. By comparing between the SOX17 + BLSE and SOX17 + LSE culture conditions, we found that genes associated with nascent amnion formation were enhanced in the presence of BMP4 (Fig. 2F,G). Thus, an important role of BMP4 is thought to be the allocation of the nascent amnionic cell lineage, which includes precursors of PGCs^6,18^. In the human PGCLC system, GATA2 and GATA3 are critical effectors downstream of BMP4, and simultaneous expression of exogenous *GATA3* and *GATA2*, in combination with *SOX17* and *TFAP2C*, has been shown to induce a robust number of human PGCLCs^19^. Consistent with these previous findings, in our present experiments *GATA2* and *GATA3* expression were enhanced in the presence of BMP4 during cjPGCLC induction. These results suggest that the functional contribution of GATA3 and GATA2 is to allocate nascent amnionic cells with competence for differentiation into PGCs.

Although the cjPGCLC system was reminiscent of the systems in humans and cynomolgus monkeys, some specific conditions for a robust induction of cjPGCLCs were found. One was the inhibition of WNT signaling during cjPGCLC induction. Without such inhibition, the expression of pluripotency-associated genes was quickly downregulated, suggesting that cjESCs after pre-treatment with a WNT agonist are sufficiently primed for differentiation. Consistent with this finding, prolonged WNT signaling has been shown to induce genes promoting EMT, the initial process of gastrulation (Fig. 3D). In the mouse PGCLC system, it was recently revealed that the expression of pluripotency-associated genes is readily attenuated by EMT-related gene expression^43^. Therefore, one of the main effects of IWR1 would be ensuring the competence of PGCLC differentiation by suppressing EMT progression.

Another specific condition for efficient cjPGCLC induction is the inhibition of retinoic acid signaling, by addition of BMS493, during cjPGCLC induction. This approach is based on the observation that WNT inhibition during cjPGCLC induction enhanced the gene expression related to neural differentiation, consistent with a well-known signaling network in which an endogenous WNT antagonist, such as DKK1, plays a central role in anteriorization of the pluripotent epiblast at the onset of gastrulation. Looking at the transcriptomic trajectory in the presence of BMS493 (Fig. 4C,D), the gene expression profile at 48 h of PGCLC induction showed a trend of transient expression of EMT-related genes, such as *VIM* and *SNAI2* (Fig. 4D). This led us to the simple idea that the addition of BMS493 attenuates the effect of IWR1, thereby creating a posterior epiblast state in which EMT and PGC specification occur simultaneously. The molecular mechanisms of crosstalk between WNT signals and retinoic acid signals during PGCLC induction are still unclear. However, previous studies have suggested that the interaction of these factors regulates the body axis formation in a stage-specific manner; for example, hyperactivation of the retinoic acid signal causes duplication of the primitive streak accompanying with expansion of *Nodal* and *T* expression^44^, and there is a mutually exclusive effect between retinoic acid and WNT signals on determination of the A–P axis during body axis extension^45^. This study will provide a useful tool to investigate the crosstalk of retinoic acid and WNT signaling during PGC induction.

Although cjPGCLCs exhibited a property of differentiated germ cells in the reaggregates with mouse gonadal somatic cells, we did not observe a robust entry into meiosis, which is a gold standard for differentiation as oocytes. Instead, cjPCLCs continued to proliferate and eventually became dominant in the reaggregates (Fig. 5B,C). This highly proliferative activity was reminiscent of human PGCLCs^46^: unlike mouse PGCLCs, which simultaneously enter meiosis within a week after reaggregation^41^, human PGCLCs keep proliferating for more than 70 days. While proliferating in the reaggregates, cjPGCLCs continuously expressed pluripotency-associated genes, such as *POU5F1* and *NANOG*, for at least 70 days of culture (Fig. 5E). Such a long-term expression of pluripotency-associated genes seems plausible, since PGCs in common marmoset embryos indeed express pluripotency-associated genes including *POU5F1*, *NANOG*, *SALL4* and *LIN28* in both male and female gonads until at least embryonic day 75^47^. In the aggregates, we observed clusters formed by several cjPGCLCs (Fig. 5B and Supplementary Fig. 4C). This histological property has also been observed in PGCs in vivo^47^. These results and other similarities to PGCs in vivo indicated that cjPGCLCs possess properties of *bona fide* PGCs. Although the expression of a meiotic gene, SYCE3, was somehow enhanced in the reaggregates, we could not detect obvious formation of synaptonemal complex. It will probably be necessary to develop further culture conditions in order to promote the differentiation into primary oocytes in culture. In parallel, it would be important to know how the onset of meiosis is regulated in PGCs during embryogenesis of the common marmoset. The optimal environment may be provided by reaggregation with marmoset gonadal somatic cells or an equivalent cell type induced from cjESCs (see below).

The multiple refinements in this study led to the identification of a culture condition by which cjPGCLCs from multiple ESC lines could be induced without genetic manipulation, and cjPGCLCs could be isolated by staining with antibodies against PDPN and KIT, followed by FACS sorting. These technical advances will expand the application of cjPGCLCs for the purpose of clarifying primate germ cell development as well as for the use of mature gametes produced in culture. Especially for the latter purpose, given the relatively short gestation and lifespan of these cells, the cjPGCLC system will provide a unique platform to validate the functionality of in vitro-made gametes, which will be a valuable model for human in vitro gametogenesis. In order to fully realize in vitro gametogenesis in the common marmoset, an inevitable obstacle for producing gametes from PGCLCs is the scarcity of syngeneic gonadal somatic cells derived from mid-gestation embryos. To overcome this obstacle, we recently established a culture system in mice that induces functional gonadal somatic cells from pluripotent stem cells^48^. Using this mouse model, the induction of gonadal somatic cells from cjESCs will become feasible, followed by the use of such cells to construct reproductive organoids in the common marmoset. This study thus provides a reliable system for the production of PGCLCs, and will ultimately contribute to the reconstitution of reproductive organs.

## Materials and methods

### Summary of cjPGCLC induction from cjESCs

cjPGCLCs were induced from cjESCs by pre-treatment followed by PGCLC induction. Briefly, cjESCs were cultured for 1 day in a pre-treatment medium (GMEM containing 15% KSR, 0.1 mM NEAA, 1 mM sodium pyruvate, 2 mM GlutaMax, 0.1 mM 2-mercaptoethanol, 1 × penicillin/streptomycin, 1 μM IM12, 50 ng/ml activin A and 10 μM ROCK inhibitor). The pretreated cells were then cultured for 4–6 days in a PGCLC induction medium (GMEM containing 15% KSR, 0.1 mM NEAA, 1 mM sodium pyruvate, 2 mM GlutaMax, 0.1 mM 2-mercaptoethanol, 1 × penicillin/streptomycin, 200 ng/ml BMP4, 100 ng/ml SCF, 1000 U/ml LIF, 50 ng/ml EGF and 10 μM ROCK inhibitor). Further details of the origins of factors and small molecules and each set of culture conditions is described below.

### cjESC lines

The cjESC line (CMES40: 46XX) was obtained from the RIKEN BioResource Center Cell Bank. This cjESC line was used for generation of the *BLIMP1*-*tdTomato* (BT) and *NANOS3-EGFP* (NG) reporter lines and generation of BTNG-cjESCs bearing inducible expression vectors for exogenous *SOX17*, *PRDM1* and *TFAP2C*. Other cjESC lines (C4: 46XX; C6: 46XX; C10: 46XX; C11: 46XY; C13: 46XX; C14: 46XX; C15: 46XX; and C17: 46XY) were established at the Central Institute for Experimental Animals^29^.

### Culture of cjESCs

All cjESC lines were maintained on irradiated mouse embryonic fibroblast (MEF) cells (3.5–4 × 10^4^ cells/cm^2^) in a humidified incubator with 5% CO_2_ and 5% O_2_ at 37 °C. The culture media used in this study were as follows: DK20FA (Knockout DMEM (Thermo, #10829018) containing 20% KSR (Thermo, #10828028), 0.1 mM NEAA (Thermo, #11140050), 1 mM GlutaMax (Thermo, #35050061), and 1xpenicillin/streptomycin (Thermo, #15070063)); 0.1 mM 2-mercaptoethanol (Thermo, #21985023) supplemented with 5 ng/ml human bFGF (Wako, #064-04541) and 10 ng/ml human/murine/rat activin A (PEPROTECH, #120-14); and PESFA (Primate ESC medium (REPROCELL, #RCHEMD001) supplemented with 5 ng/ml bFGF and 10 ng/ml activin A). The culture medium was changed every day.

cjESCs were passaged through either small cell clumps or single cell suspensions. For passage through small clumps, cjESCs were incubated with CTK solution (PBS containing 20% KSR, 1 μM CaCl_2_, 0.1 mg/ml collagenase type IV (Thermo, #17104019), and 0.025% trypsin EDTA (Thermo, #15090046)) for 6~10 min, washed twice with Wash medium (DMEM/Ham’sF-12 (Nacalai, #08460-95) containing 0.1% BSA (Thermo, #15260037)), and then dissociated mechanically by gentle pipetting. After centrifugation (270 g, 3 min), cell clumps were resuspended with culture medium and spread at the rate of 1:2 to 3 times dilution. This passage was done every 3 to 4 days. For passage through the single cell suspension, cjESCs were incubated with Accutase (Nacalai, #12679-54) for 5 min, supplemented with Wash medium and then dissociated by gentle pipetting. After centrifugation (270 g, 3 min), the cells were resuspended with culture medium and spread at a density of 8.0 × 10^4^ cells per 6-well plate. This passage was done every 4 to 6 days. For each passage, 10 μM Y-27632 (Wako, #034-24024) was added for 24 h after plating the cells.

### Pre-treatment of cjESCs

For the pre-treatment, cjESCs were dissociated to single cells by incubation with Accutase for 5 min and then plated on a 12-well plate coated with fibronectin (Millipore, #FC010) at a density of 2 × 10^5^ cells per well and cultured for 1 day in GK15 medium containing 1 μM IM12 (ENZO, #BML-WN102-0005), 50 ng/ml activin A and 10 μM ROCK inhibitor. After the preculture, cells were dissociated by incubation with Accutase for 5 min and then subjected to PGCLC induction.

### cjPGCLC induction

For PGCLC induction, cells were dissociated by incubation with Accutase and then transferred into a low-cell-binding U-bottom 96-well plate (Greiner, #650970) in GK15 medium (GMEM (Thermo, #11017035) containing 15% KSR, 0.1 mM NEAA, 1 mM sodium pyruvate (Thermo, #11360070), 2 mM GlutaMax, 0.1 mM 2-mercaptoethanol, and 1 × penicillin/streptomycin) supplemented with 200 ng/ml human BMP4 (R&D Systems, #314-BP-01 M), 100 ng/ml mouse SCF (R&D Systems, #455-MC-500), 1000 U/ml human LIF (Wako, #125-06661), 50 ng/ml human EGF (R&D Systems, #236-EG) and 10 μM ROCK inhibitor with or without 2.5 μM IWR1 (TOCRIS, #3532) and 1.0 µM BMS493 (R&D Systems, #3509/10). In each well, 5 × 10^3^ cells were used. In the experiments requiring enforced expression of the transgenes, 100 μM dexamethasone, 0.5 μg/ml doxycycline, and 0.5 μM Shield-1, which induce *SOX17*, *BLIMP1*, and *TFAP2C* expression, respectively, were added to the medium^11^. To enforce the expression of SOX17 alone, only 100 μM dexamethasone was added.

### Generation of the BTNG-reporter cjESCs

For construction of the *BLIMP1-p2A-tdTomato* and *NANOS3-p2A-eGFP* vectors for targeted insertion, the homology arms of *BLIMP1* (979 bp upstream and 1,027 bp downstream of the stop codon) and *NANOS3* (979 bp upstream and 1,106 bp downstream of the stop codon) were amplified by PCR using the primers listed in Supplementary Table 1. To nullify Cas9-mediated deletion of the targeting vector, the primer for amplification of the homology arm included a mutated PAM sequence. As backbone vectors, human *BLIMP1-p2A-tdTomato* and human *NANOS3-p2A-eGFP*^10^ were provided by Professor M. Saitou (Kyoto University). The amplified left or right arms were replaced with those in the backbone vectors by using an In-Fusion® HD Cloning Kit (Takara, #Z9649N) after treatment with the restriction enzymes (human *BLIMP1* left arm: SacI/NheI; human *BLIMP1* right arm: EcoNI/NotI; human *NANOS3* left arm: NotI/NheI; human *NANOS3* right arm: AscI/SacI). For construction of the guide RNA (gRNA)-expression vectors, complementary oligonucleotides including the guide RNA sequence close to the stop codon of *BLIMP1* or *NANOS3* were annealed and then inserted into the BbsI site of the pX330 vector (Addgene, #42,230) using a Ligation-High kit (TOYOBO, #LGK-101). All oligonucleotides used for the construction of gRNA-expression vectors are listed in Supplementary Table 1.

The targeting vectors and the gRNA-expression vectors were transfected into CMES40 cjESCs by Lipofectamine® 2000 (Nacalai, #11668-019) according to the manufacturer’s protocol. Briefly, the cjESCs were dissociated into single cells by incubation with Accutase, resuspended in DK20F (DK20FA without activin A) supplemented with 500 μL DNA-lipofectamine complex containing 2.5 μg for each targeting vector, 2.5 μg for each gRNA expression vector, 10 μl lipofectamine, and 10 μM Y-27632, and then plated on a 6-well plate coated with drug-resistant MEFs. The medium was changed to DK20F containing 200 μg/ml G418 or 500 ng/ml puromycin at 24 h after the lipofection. At 7 to 10 days of culture, single colonies were picked up and cultured on MEFs in DK20F. The targeting integrations were assessed by PCR using the primers listed in Supplementary Table 1. The cjESC lines bearing the targeted allele were used for the transfection of Cre- and GFP-expressing vectors to excise the PGK-Neo or PGK-Puro cassettes. At 2 days after the additional transfection, GFP-positive cells were sorted and then plated with DK20F supplemented with 10 µM Y-27632 at a density of 2000 cells per 6-well plate coated with MEFs. At around eight days after the cell sorting, single colonies were picked up and cultured on MEFs in DK20F. The excision of the PGK-Neo or the PGK-Puro cassettes was assessed by PCR using the primers listed in Supplementary Table 1.

### Generation of drug-inducible transgenic cjESC lines

Expression vectors for the generation of cjESCs exogenously expressing human *SOX17*, *BLIMP1*, and *TFAP2C* were provided by Dr. T. Kobayashi (The University of Tokyo)^11^. These vectors were transfected with the EF1α-dsRed expression vector into the BTNG-reporter cjESC line by lipofectamine as described above. The DNA-lipofectamine complex was composed of 0.31 μg each of *SOX17*, *BLIMP1*, *TFAP2C*, or *TET3G* expression vector, 1.5 μg piggyBac transposase expression vector, 0.27 μg EF1α-dsRed expression vector and 6 μl lipofectamine in a total solution of 300 µL. The TET3G expression vector was used to induce expression of exogenous *BLIMP1* and the neomycin-resistance gene. Single cell-suspended BTNG-reporter cjESCs were cultured in DK20F supplemented with 10 µM Y-27632 and the DNA-lipofectamine complex on MEF. The medium was exchanged with fresh DK20F at 1 day after the transfection. At 2 days of culture, dsRed-positive cells were sorted and then plated with DK20F at a density of 2000 to 3000 cells on a 6-well plate coated with MEFs. At 1 day after sorting, 200 μg/ml G418 was added for drug selection and then the emerging colonies were picked up. The integrations of *SOX17*, *BLIMP1*, and *TFAP2C* expression vectors were assessed by PCR using the primers listed in Supplementary Table 1.

### Fluorescence activated cell sorting (FACS)

To prepare the single cell suspension of cjPGCLCs, the aggregates were treated with 0.05% Trypsin–EDTA (Thermo, #15400054) in PBS for 15 min at 37 °C, dissociated by pipetting, and then filtrated through a 70 μm-pore nylon membrane. The cells were collected by centrifugation (270 g, 3 min), suspended in FACS buffer (0.1% BSA in PBS) and then analyzed and/or sorted by FACSAria Fusion (BD Biosciences). For the analysis of surface marker proteins, a single cell suspension of cjESCs or cjPGCLCs was stained with antibodies in FACS buffer for 15 min at 4 °C. The primary and secondary antibodies used in this study are listed in Supplementary Table 2.

### Culture of xenogeneic reconstituted ovaries

For the preparation of xenogeneic reconstituted ovaries, embryonic mouse gonadal somatic cells were collected as described previously^49^. Briefly, female gonads were obtained from embryonic day (E) 12.5 embryos derived from intercrosses of ICR mice. Female gonads were surgically separated from the mesonephros and then dissociated by incubation with 0.05% Trypsin–EDTA in PBS for 10 min at 37 °C. Endogenous PGCs in the dissociated gonadal cells were removed by MACS using anti-SSEA1 and anti-CD31 antibody conjugated with magnetic beads (Miltenyi Biotech)*.* The dissociated gonadal somatic cells were reaggregated with FACS-sorted cjPGCLCs. Culture of xenogeneic reconstituted ovaries was performed as previously reported with slight modifications^41^. Reaggregations containing 500 cjPGCLCs at day 6 and 37,500 E12.5 female gonadal somatic cells were cultured in a low-binding U-bottom 96-well plate for 2 days in GK15 medium supplemented with 1 μM retinoic acid and 10 μM ROCK inhibitor. After this period, the xenogeneic reconstituted ovaries were placed on Transwell-COL membranes (CORNING, #3492) soaked in αMEM (Thermo, #12571063) containing 2% fetal calf serum (FCS), 55 μM 2ME, 1xpenicillin/streptomycin, 1xGlutaMAX, and 150 μM ascorbic acid. At 4 days of culture, the medium was changed to S10 medium (StemPro (Thermo, #10639011) containing 10% FCS, 55 μM 2ME, 1xpenicillin/streptomycin, 1xGlutaMAX, and 150 μM ascorbic acid). The medium was changed every other day.

For analysis of xenogeneic reconstituted ovaries, the reconstituted ovaries were treated with CTK solution for 30 min at 37 °C, transferred into Accutase and then incubated at 37 °C for 15 min. After these incubations, the xenogeneic reconstituted ovaries were dissociated and then filtrated with a 70 μm-pore nylon filter. The dissociated cells were collected by centrifugation and suspended in FACS buffer for analysis.

### Immunofluorescence analysis

For immunofluorescence analysis of cjESCs, cells were cultured on an ibidi μ-Slide 8 Well (ibidi, #80826) coated with MEF in DK20FA or PESFA. These cjESCs were washed once with PBS, fixed with 4% paraformaldehyde (PFA) in PBS for 15 min at room temperature, washed twice with PBST (PBS containing 0.1% Tween 20), permeabilized with 0.2% Triton X in PBS for 15 min at room temperature, and then washed twice with PBST for the following processes. The slides were incubated with Blocking One Histo (Nacalai, #03953-95) for 10 min at room temperature, washed once with PBST, and incubated overnight at 4 °C with primary antibodies in Blocking One Histo that was 20-fold diluted in PBST. After washing three times with PBST, the slides were incubated for 2 h at room temperature with secondary antibodies and 1 μg/ml of DAPI in Blocking One Histo that was 20-fold diluted in PBST. After washing three times with PBST, the slides were mounted with Fluoro-KEEPER Antifade Reagent (Nacalai, #12593-64), and analyzed using an LSM700 laser microscope (ZEISS).

For immunofluorescence analysis of cjPGCLCs, BTNG-positive cells were sorted by FACSAria Fusion. As a control, cjESCs were sorted after staining with anti-human TRA-1-60-R conjugated with PE to separate them from MEFs. The sorted BTNG-positive cells and TRA-1-60-R-positive cjESCs were mixed at a ratio of 1:1–1:2 and suspended at a concentration of 1–2 × 10^5^ cells/ml with PBS containing 1%BSA. One hundred microliters of the cell suspension were spread onto MAS-coated glass slides (Matsunami, #MAS-04) using a CytoSpine4 (Thermo) at 800 rpm for 3 min. The slides were then fixed with 4% PFA in PBS for 15 min at room temperature, washed twice with PBS and once with PBST, permeabilized with 0.5% Triton X in PBS for 10 min at room temperature, and washed three times with PBS for the following processes. The slides were incubated with Blocking One Histo for 10 min at room temperature and washed once with PBS. Next, the slides were incubated with primary antibodies in Blocking One Histo that was 15-fold diluted in PBST overnight at 4 °C followed by washing twice with PBST and twice with PBS. The slides were then incubated with secondary antibodies and 1 μg/ml of DAPI in Blocking One Histo that was 15-fold diluted in PBST for 3 h at room temperature. After washing twice with PBST and twice with PBS, the sections were mounted with PermaFluor (Thermo, #TA-030-FM) and analyzed using an LSM700 laser microscope. For the immunofluorescence of 5mC, the slides were treated with 4N HCl in 0.1% Triton X for 10 min at room temperature and neutralized with 10 mM Tris–HCl (pH 8.0) for 10 min followed by washing twice with PBS and, finally, treatment with Blocking One Histo.

For immunofluorescence analysis of xenogeneic reconstituted ovaries, the samples were stripped from Transwell-COL membranes using micro forceps. Then, the samples were fixed with 4% PFA in PBS for 3 h at 4 °C, soaked sequentially in 10%, 15%, and 20% sucrose and then treated in fresh 20% sucrose overnight at 4 °C. The samples were then embedded in O.C.T. compound (Tissue-Tek, #4583) and sectioned at a thickness of 7 μm. Air-dried sections were washed three times with PBS, incubated with Blocking One Histo for 1 h at room temperature and washed once with PBS for the following processes. The sections were incubated with primary antibodies in Blocking One Histo that was 15-fold diluted in PBST overnight at 4 °C, washed twice with PBST and once with PBS, and then incubated with secondary antibodies and 1 μg/ml of DAPI in the same solution for the first antibody reaction for 3 h at room temperature or overnight at 4 °C. After washing twice with PBST and once with PBS, the sections were mounted with PermaFluor, and analyzed using an LSM700 laser microscope. The primary and secondary antibodies used in this study are listed in Supplementary Table 2.

### Transcriptome analysis

For transcriptome analysis, 10,000 cells for each sample were collected by FACSAria Fusion (BD Biosciences). All samples used for transcriptome analysis are listed in Supplementary Table 3. Biologically duplicated samples were prepared under each condition. Total RNAs were extracted using an RNeasy Micro Kit (QIAGEN #74,004), and poly(A)^+^ RNAs were purified using a NEBNext Poly(A) mRNA Magnetic Isolation Module (New England Biolabs #E7490). Directional RNA sequence libraries were constructed using a NEBNext Ultra Directional RNA Library Prep Kit for Illumina (New England Biolabs #E7760) as described previously^50^. cDNAs were enriched by 12-cycle PCR. HiSeq2000 (Illumina) was used to perform single-end sequencing. Read quality was assessed by FastQC 0.11.9 (https://github.com/s-andrews/FastQC). RNA-seq data of the blastocysts and epiblasts of Callithrix Jacchus and Naïve state cjESCs were obtained from GSE138944, and poor-quality reads (score < 30) were trimmed with Trim Galore! (https://github.com/FelixKrueger/TrimGalore) as described previously^31^. RNA-seq data of the naïve and primed state human ESCs were obtained from E-MTAB- 5674^30^, and poor-quality reads (quality value < 20, read length < 20) were trimmed with fastp 0.20.0. To minimize technical variability, the sequencing modes were truncated to single-end format. Reads from each species were aligned to GRCh38/hg38 for human and Callithrix_jacchus_cj1700_1.1 for Callithrix jacchus with STAR 2.7.7a^51^. Alignments to gene loci were filtered for MAPQ values > 1 (maps to less than 9 locations in the target) with samtools 1.10^52^. Filtered alignments were quantified with rsem 1.3.3^53^ based on annotation from GRCh38/hg38 for human and Callithrix_jacchus_cj1700_1.1 for Callithrix jacchus. The expression levels were normalized by TPM.

The annotation from Callithrix_jacchus_cj1700_1.1 contains many genes of uncertain function (LOC symbols), and therefore common genes (15,755 genes: including POU5F1) between Callithrix jacchus and humans were used. The common genes were defined as described in the “Common genes listed in the marmoset–human one-to-one annotation table” section.

For principal component analysis (PCA), the analysis of differentially expressed genes (DEGs), and unsupervised hierarchical clustering (UHC) analysis, we used the genes selected by log_2_(TPM + 1) ≧1.5 in at least one sample (11,398 genes: common genes and log_2_(TPM + 1)≧1.5). PCA was performed using R software with FactoMineR v.2.3^54^ based on log_2_(TPM + 1). UHC analysis was performed using R software with the hclust function with Euclidian distances and Ward’s method (ward.D2). For identification of DEGs, the false discovery rate (FDR), log_2_ CPM and log_2_ fold-change were calculated using edgeR v.3.28.0^55^. The DEGs were defined as genes exhibiting a more than four-fold difference between the samples (FDR <  0.01 and log_2_CPM > 1.5 as the mean of the expression level in the group). The DAVID 2021 was used for gene ontology (GO) analysis^56^. Since the annotation for late germ cell development in the common marmosets was insufficient, the GO analysis for xenogeneic reconstituted ovaries was performed by the annotations in humans using the common genes between marmosets and humans (see below).

### Common genes listed in the marmoset–human one-to-one annotation table

In order to analyze RNA sequence data except for the effect of genes with uncertain function (LOC symbols) in marmosets, we made a Common genes listed in the marmoset–human one-to-one annotation table by means of a genomic coordinate comparison using the LiftOver tool, as described previously^10,57^. There are 21,504 genes in Callithrix_jacchus_cj1700_1.1 and 17,351 in GRCh38/hg38, with 15,754 genes in common between them (Supplementary Table 4). Since *POU5F1* was not included among the common genes, it was added to the table manually, resulting in a total of 15,755 genes.

## Supplementary Information

Supplementary Information 1.

Supplementary Information 2.

Supplementary Information 3.

Supplementary Information 4.

Supplementary Information 5.

Supplementary Information 6.

## Data Availability

The RNA-seq and methylome data have been deposited at the Gene Expression Omnibus (GEO) database under the accession number GSE201320.
