# Supplementary material for: Induction of primordial germ cell-like cells from common marmoset embryonic stem cells by inhibition of WNT and retinoic acid signaling
Source: Sci Rep. 2023 Feb 23;13:3186. doi: 10.1038/s41598-023-29850-z (PMC9950483; doi:10.1038/s41598-023-29850-z)
Supplement: Supplementary file 1 — Supplementary Information 1. [file 41598_2023_29850_MOESM1_ESM.docx]

**Supplementary Figure 1. Production and validation of reporter cjESCs.**

**(A)** Immunofluorescence analysis of pluripotency-associated gene expression. Images show the expression of POU5F1 (green), SOX2 (red), and NANOG (red) with DAPI (white) in BTNG-cjESCs cultured in DK20FA or PESFA. Scale bar, 100 μm. Histograms below the images show the expressions of INTEGRINA6, TRA1-60, and SSEA4 in cjESCs cultured in DK20FA or PESFA. **(B)** Proliferation rates of cjESC clones cultured on MEF in PESFA and in DK20FA. cjESCs were passaged through a single cell suspension every 4 to 6 days (red and blue) or small clumps every 3 to 4 days (grey). **(C)**Targeted insertion of the *BLIMP1*-*tdTomato* (BT) and *NANOS3*-*EGFP* (NG) reporter constructs. Black boxes and asterisks indicate exons and the gRNA-recognition site for the CRISPR Cas9 system, respectively. The images show the results of PCR for genotyping. The circle above the gel image indicates the ESC clone used in this study. Sequences of the primers are available in Supplementary Table 1. M, size marker.

**Supplementary Figure 2. Cell populations used for the RNA-seq analyses in this study**

**(A-C)** Cell population used for the RNA-seq analyses. FACS plots show the cells at each time point in culture under the condition indicated. Cells enclosed by a dashed red line were used for the RNA-seq analyses shown in Figure 2 (A), Figure 3 (B) and Figure 4 (C).

**Supplementary Figure 3. Differentially Expressed Genes in cells cultured with IWR1 and BMS493.**

**(A)** GO analysis of genes affected by IWR1. The scatterplots show the genes differentially expressed between BLSE+IWR1 and BLSE (Log_2_fold-change > 2, Log_2_averageCPM > 1.5, and false discovery rate (FDR) < 0.01) at 48 hr of induction. Representative GO terms for each gene group and their p-values are shown at right. **(B)** Scatter plot comparison between GO analysis of BTNG-positive cells. Shown are scatter plots of transcriptomes of OGBT-positive cells at 96 hr of culture indicated with DEGs (Log_2_fold-change > 2, Log_2_averageCPM > 1.5, and FDR < 0.01). Comparison with the transcriptome of total cell population at 96 hr of BLSE culture is shown as a reference.

**Supplementary Figure 4. Reproducibility of epigenetic and differentiation properties in BTNG-positive cells induced with IWR1 and BMS493.**

**(A)** Immunofluorescence analyses of representative epigenetic markers in cjPGCLCs induced with BMS493. Images show the results of immunofluorescence analysis of epigenetic markers indicated with DAPI in SOX17(+) cjPGCLCs at day 4 of culture under the BLSERI+BMS condition. Plots on the right show relative fluorescence intensities, in comparison to those in cjESCs, determined by the Image J software. The average fluorescence intensity of cjESCs was set as 1. ***P<0.001 (using Welch’s t test). NS, not significant. Scale bar, 20 μm. **(B)** A schematic experimental procedure for the construction and culture of the xenogeneic reconstituted ovary. **(C)** Expression of DDX4 in the reaggregations with mouse gonadal somatic cells. Images show the results of immunofluorescence analysis of DDX4 with DAPI in BT(+) cjPGCLCs induced with BMS493 in the xenogeneic reconstituted ovaries at day 40, day 70, and day 90 of culture. Scale bars, 20 μm.

**Supplementary Figure 5. Expression of surface marker proteins and non-reporter cjESC lines used in this study.**

**(A)** FACS analysis of surface marker proteins for isolation of cjPGCLCs. FACS plots show the expressions of PDPN, KIT, and ITGA6 on cells at day 4 of cjPGCLC induction by exogenous SOX17 expression under the BLSER condition. **(B)** Transcripts of genes encoding the surface marker proteins. Each value is based on duplicated RNA-seq analyses. **(C)** Comparison of the proliferation rates of multiple cjESC lines cultured on MEF in PESFA. Cells were passaged through a single cell suspension every 4 to 6 days of culture.
