# Supplementary material for: Induction of primordial germ cell-like cells from common marmoset embryonic stem cells by inhibition of WNT and retinoic acid signaling
Source: Sci Rep. 2023 Feb 23;13:3186. doi: 10.1038/s41598-023-29850-z (PMC9950483; doi:10.1038/s41598-023-29850-z)

(Supplementary Figure 1C)

PRDM1(BLIMP1) locus

Left arm

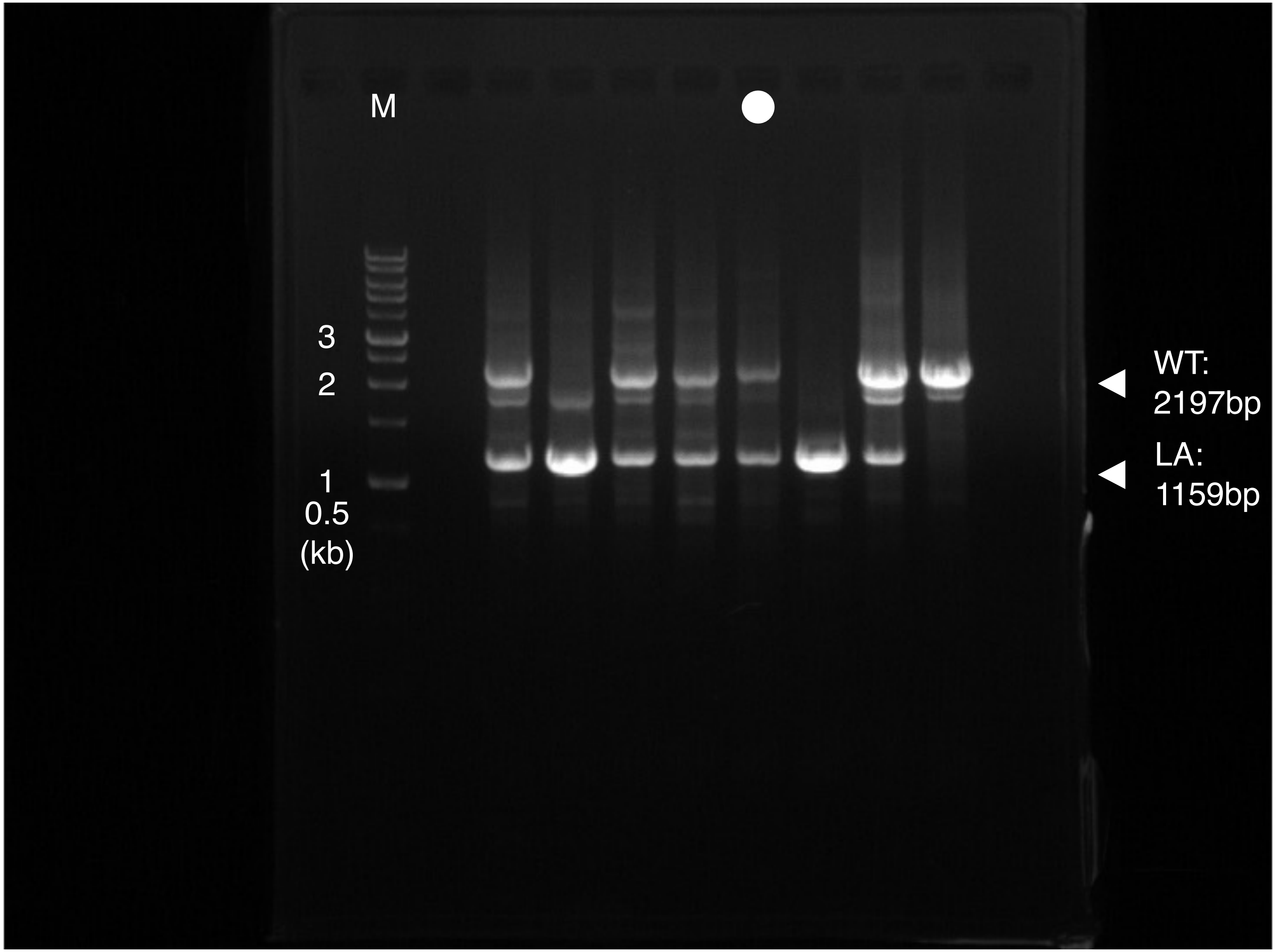

(Supplementary Figure 1C)

PRDM1(BLIMP1) locus
Right arm

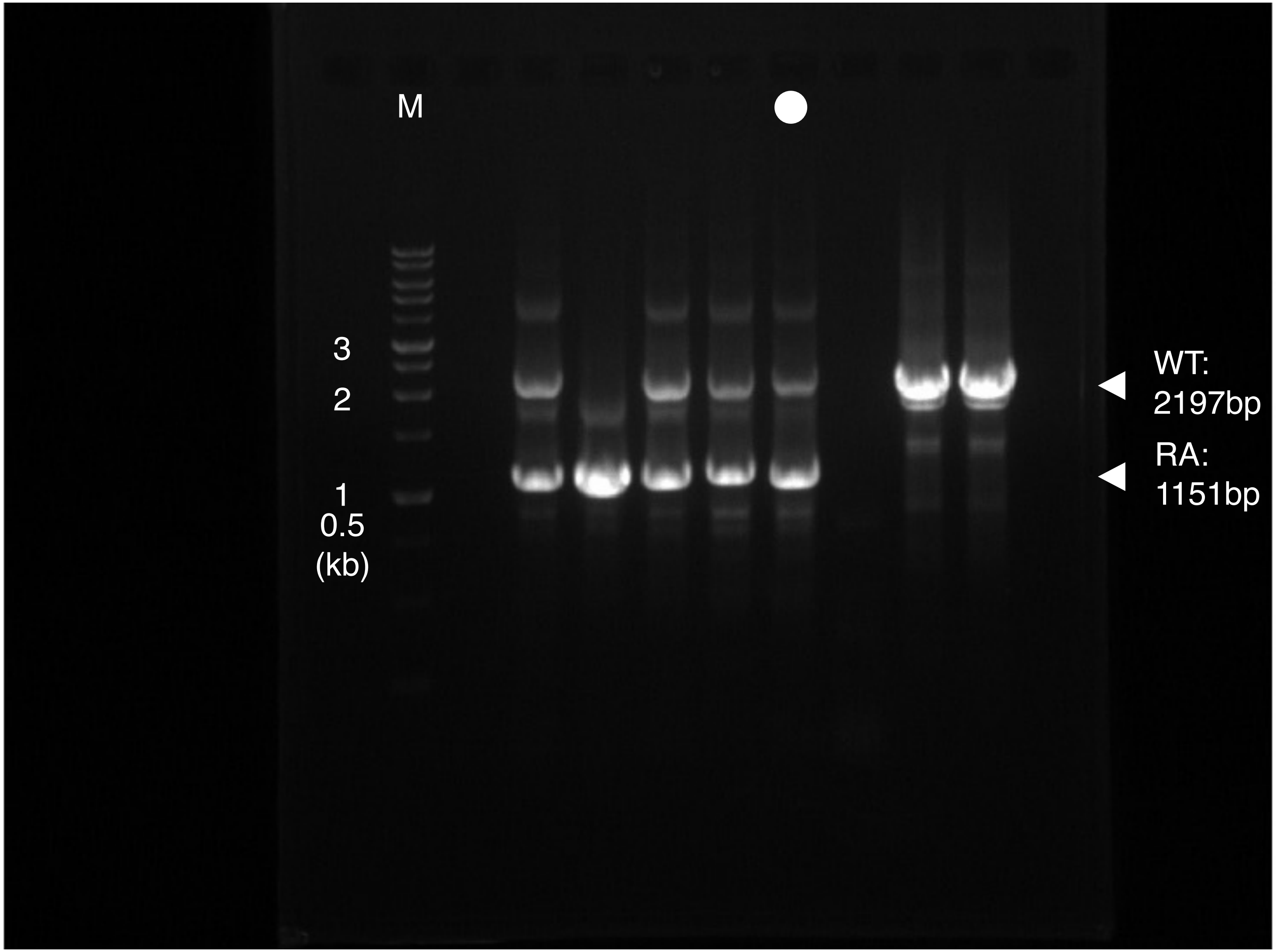

(Supplementary Figure 1C)

PRDM1(BLIMP1) locus
Δlox

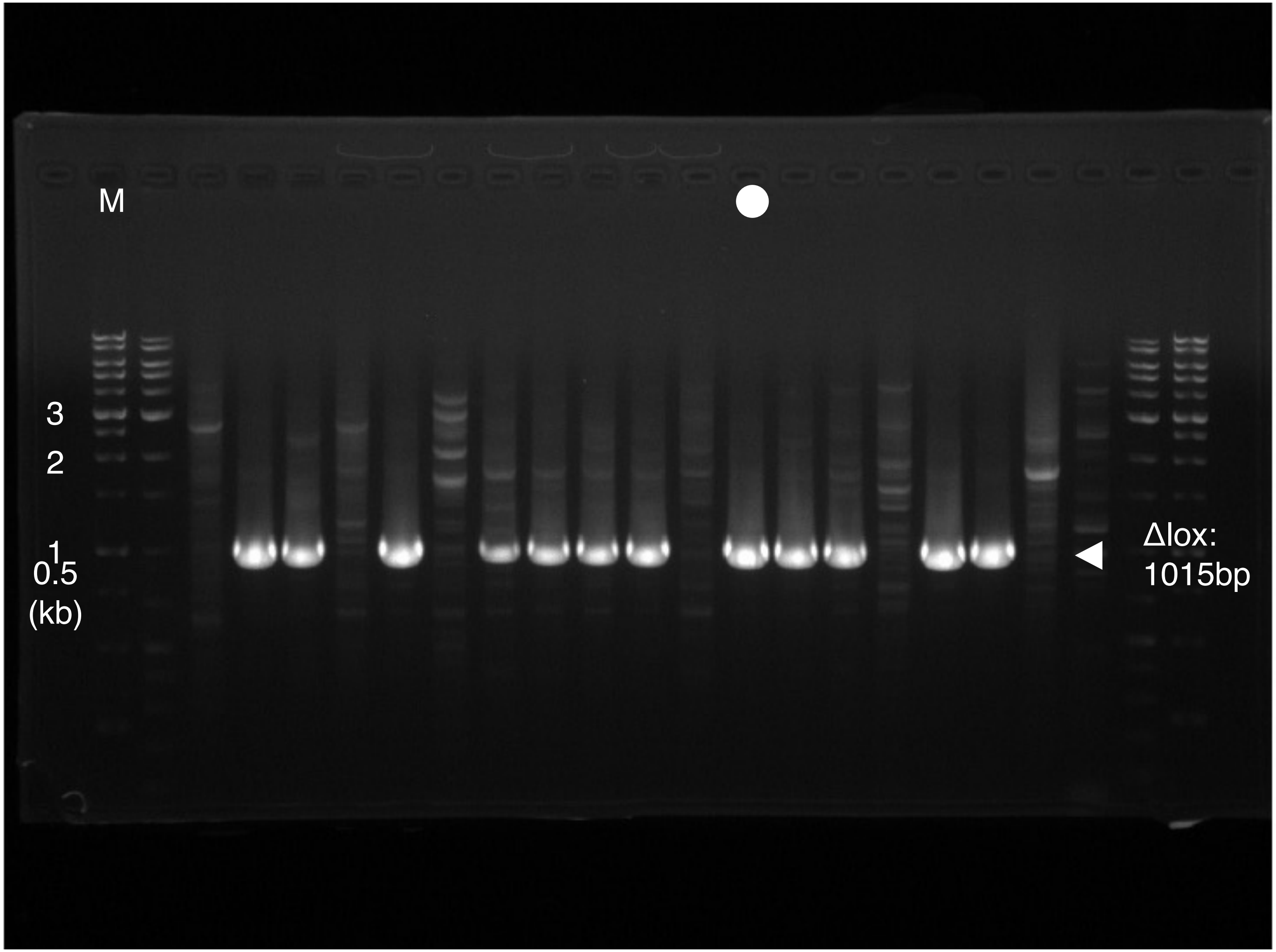

(Supplementary Figure 1C)

NANOS3 locus

Left arm

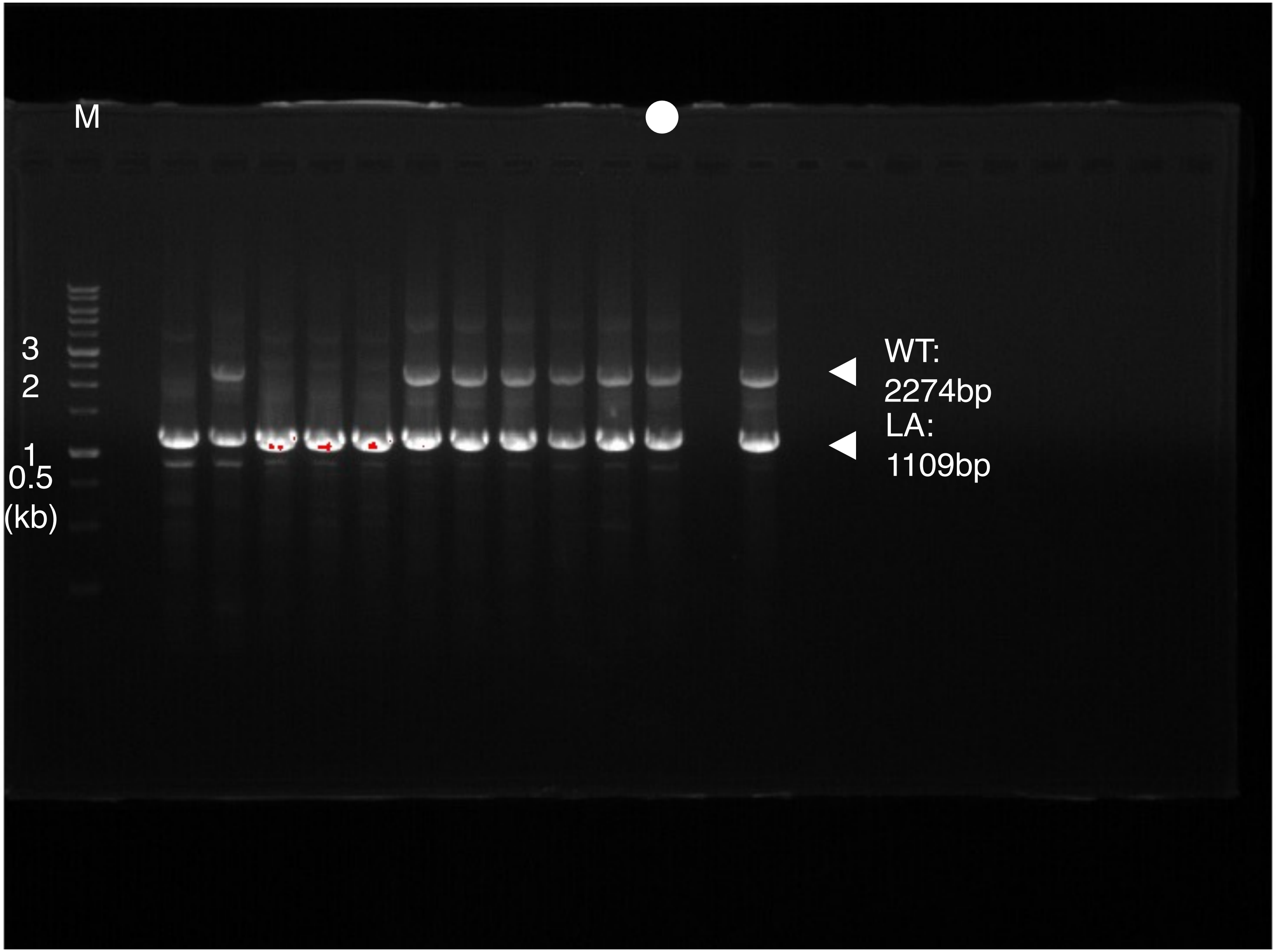

(Supplementary Figure 1C)

NANOS3 locus

Right arm

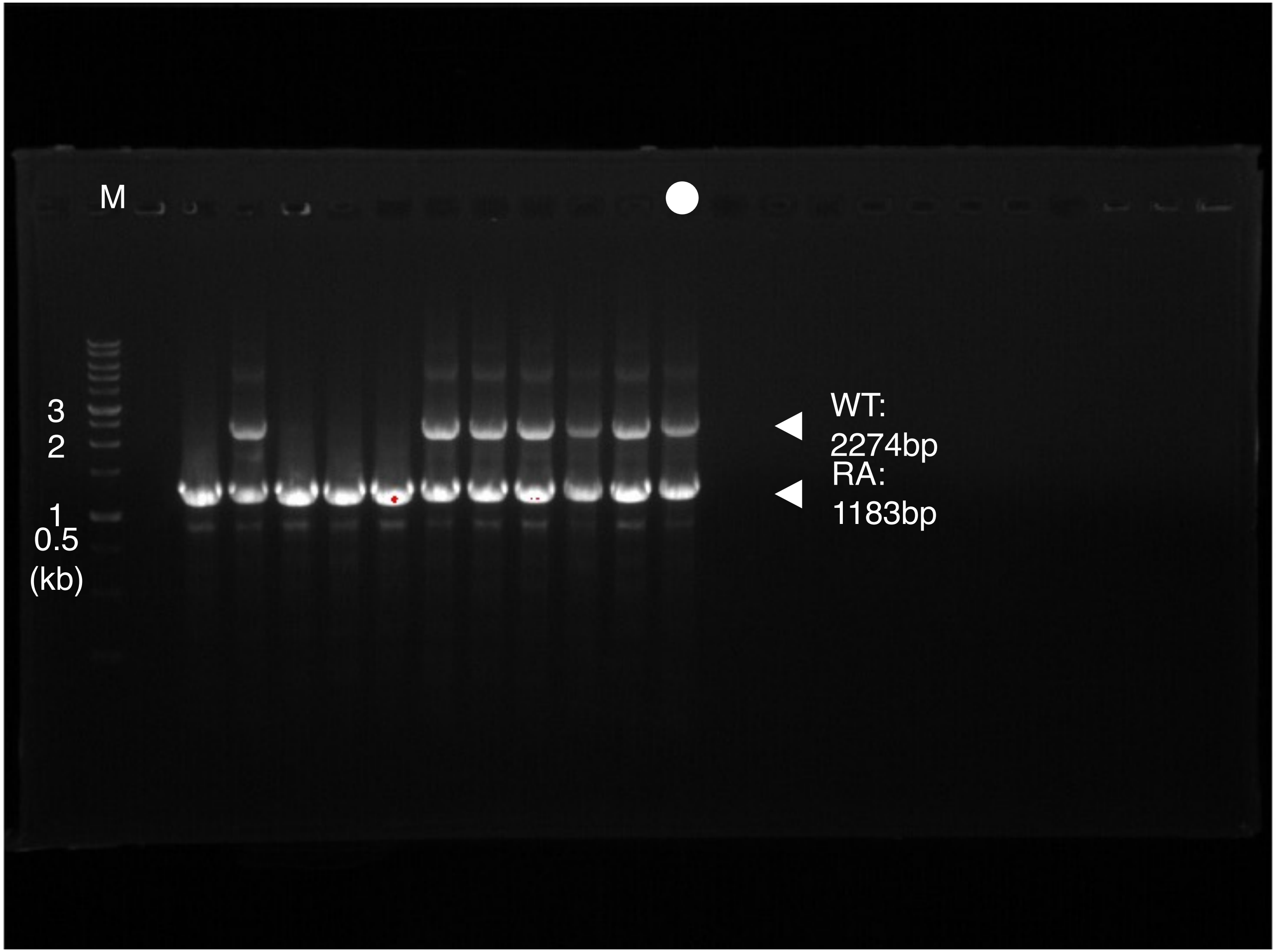

(Supplementary Figure 1C)

NANOS3 locus

Δlox

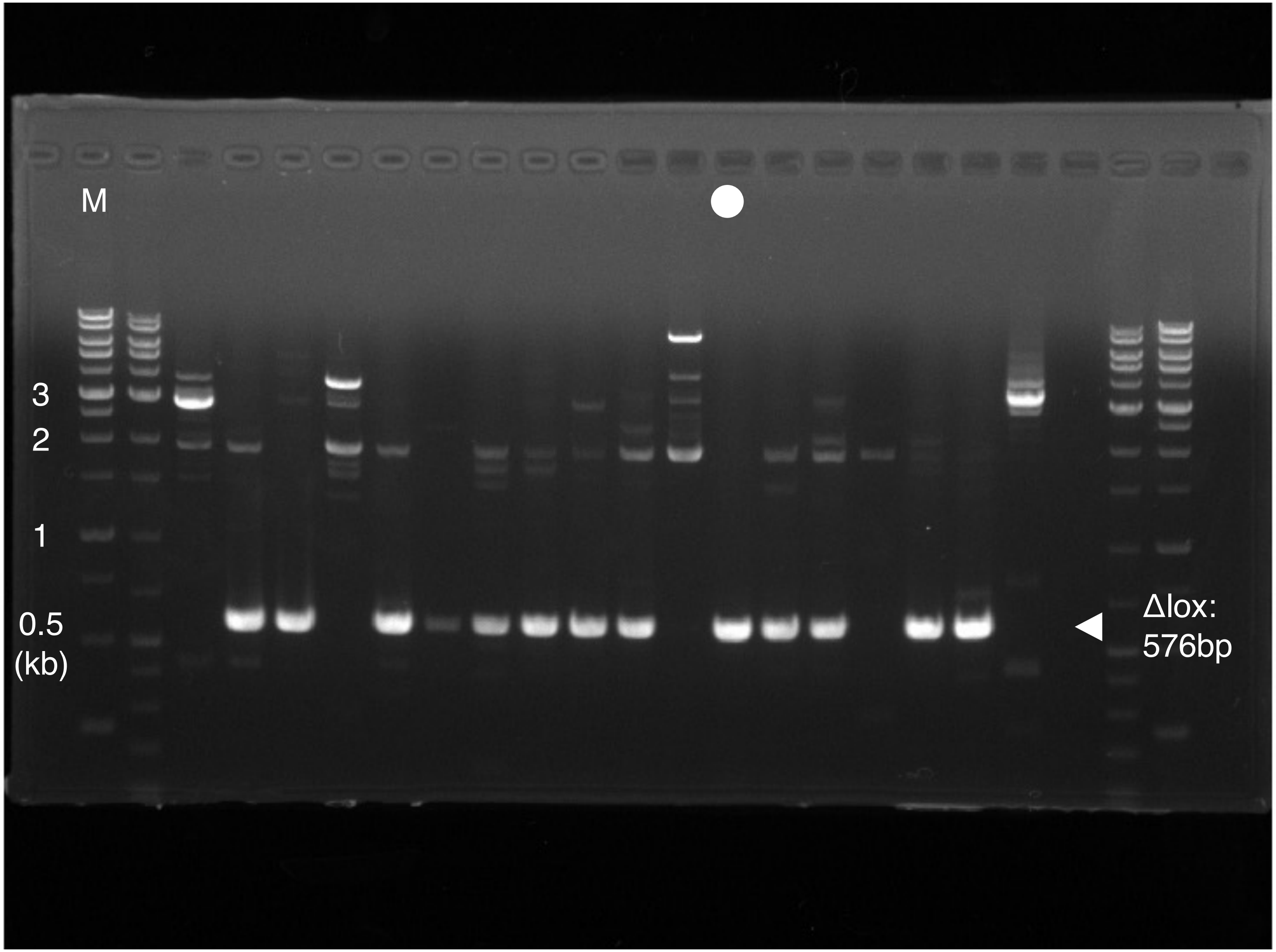

Supplement: Supplementary file 6 — Supplementary Information 6. [file 41598_2023_29850_MOESM6_ESM.pdf]
